# Concomitant Coracoid Process Fracture and Massive Rotator Cuff Tear: A Rare Case Report and Surgical Management Approach

**DOI:** 10.7759/cureus.81709

**Published:** 2025-04-04

**Authors:** Dimitrios Giotis, Christos Konstantinidis, Sotiris Plakoutsis, Dimitrios Vardakas, Alkisti Konstantinou

**Affiliations:** 1 Department of Orthopaedics, General Hospital of Ioannina "G. Hatzikosta", Ioannina, GRC

**Keywords:** coracoid process fracture, deltopectoral approach, high-energy trauma, rotator cuff tear, surgical management

## Abstract

Coracoid process fractures are rare injuries that can be overlooked, especially when they occur in isolation or alongside soft tissue damage. This case report describes a unique clinical presentation involving a displaced coracoid process fracture with a concomitant massive rotator cuff tear following high-energy trauma. The diagnostic process required a multimodal imaging approach to accurately assess both bony and soft tissue structures, which was critical in formulating an effective treatment plan. Surgical management involved open reduction and internal fixation of the coracoid fracture through a deltopectoral approach, followed by a mini-open lateral approach for rotator cuff repair using suture anchors. Postoperative rehabilitation was structured in progressive phases to support tendon healing and functional recovery. The patient achieved full, pain-free range of motion and radiographic union by six months post-surgery. This case underscores the need for heightened clinical awareness when evaluating anterior shoulder trauma and demonstrates that a dual surgical approach, tailored to address both osseous and soft tissue injuries, can yield excellent outcomes. Reporting such rare cases contributes to the evolving understanding and management strategies for complex shoulder injuries involving the coracoid process and rotator cuff.

## Introduction

Fractures of the coracoid process are rare orthopedic injuries, accounting for less than 1% of all fractures and approximately 2-13% of scapular fractures [1,2]. They typically result from high-energy trauma, such as motor vehicle accidents or falls from significant heights [2]. Isolated coracoid fractures are uncommon and are often accompanied by other musculoskeletal injuries, which can lead to delayed or missed diagnoses [2]. Given the anatomical proximity to critical soft tissue structures, including the labrum, rotator cuff, brachial plexus, and nearby neurovascular elements, a thorough evaluation of both bony and soft tissue components is necessary [3,4].

Clinical evaluation alone may not be sufficient due to overlapping symptoms [2]. Radiographic imaging is the initial modality for fracture assessment. However, computed tomography (CT) scan is often required when the diagnosis is unclear [5]. Magnetic resonance imaging (MRI) is essential to evaluate associated soft tissue injuries, particularly rotator cuff tears [2,4]. Fractures of the coracoid process can be associated with acromioclavicular or glenohumeral dislocations, fractures of the scapular body, clavicle, or proximal humerus [1,3,5].

While isolated cases of either coracoid process fractures or rotator cuff injuries have been well-documented, the simultaneous presentation of both is exceptionally uncommon [5-7]. The purpose of this study is to present a rare case of a displaced coracoid process fracture concomitant with a massive rotator cuff tear, successfully managed with open reduction and internal fixation (ORIF) along with open rotator cuff repair. The diagnostic challenges and management considerations associated with this rare combination are also highlighted.

## Case presentation

A 56-year-old male patient with an unremarkable medical history presented to the emergency department of our hospital with a right shoulder injury after falling from a height of three meters. He reported a direct impact to the anterior aspect of the shoulder upon striking the ground. Upon arrival, he was hemodynamically stable, with a normal blood pressure of 110/71 mmHg, regular heart rate of 95 beats per minute, and a respiratory rate of 17 breaths per minute.

**Figure 1:**
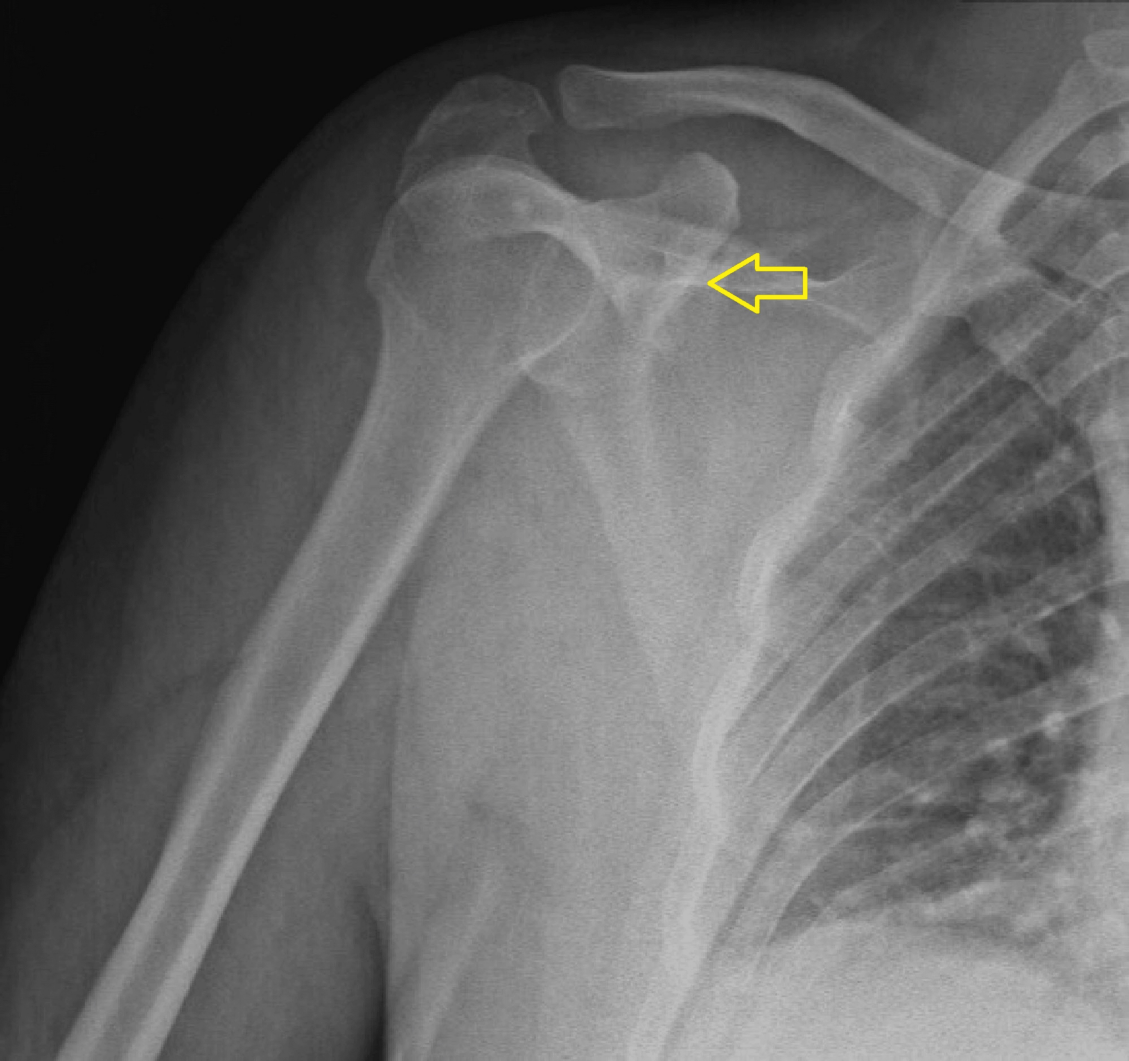
Preoperative X-ray of the right shoulder joint showing suspicion of a coracoid process fracture (yellow arrow).

**Figure 2:**
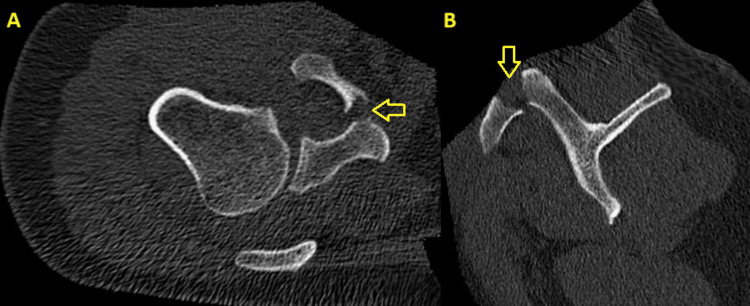
Preoperative CT scan. The yellow arrows highlight the displaced coracoid process fracture. (A) Transverse view. (B) Sagittal view.

**Figure 3:**
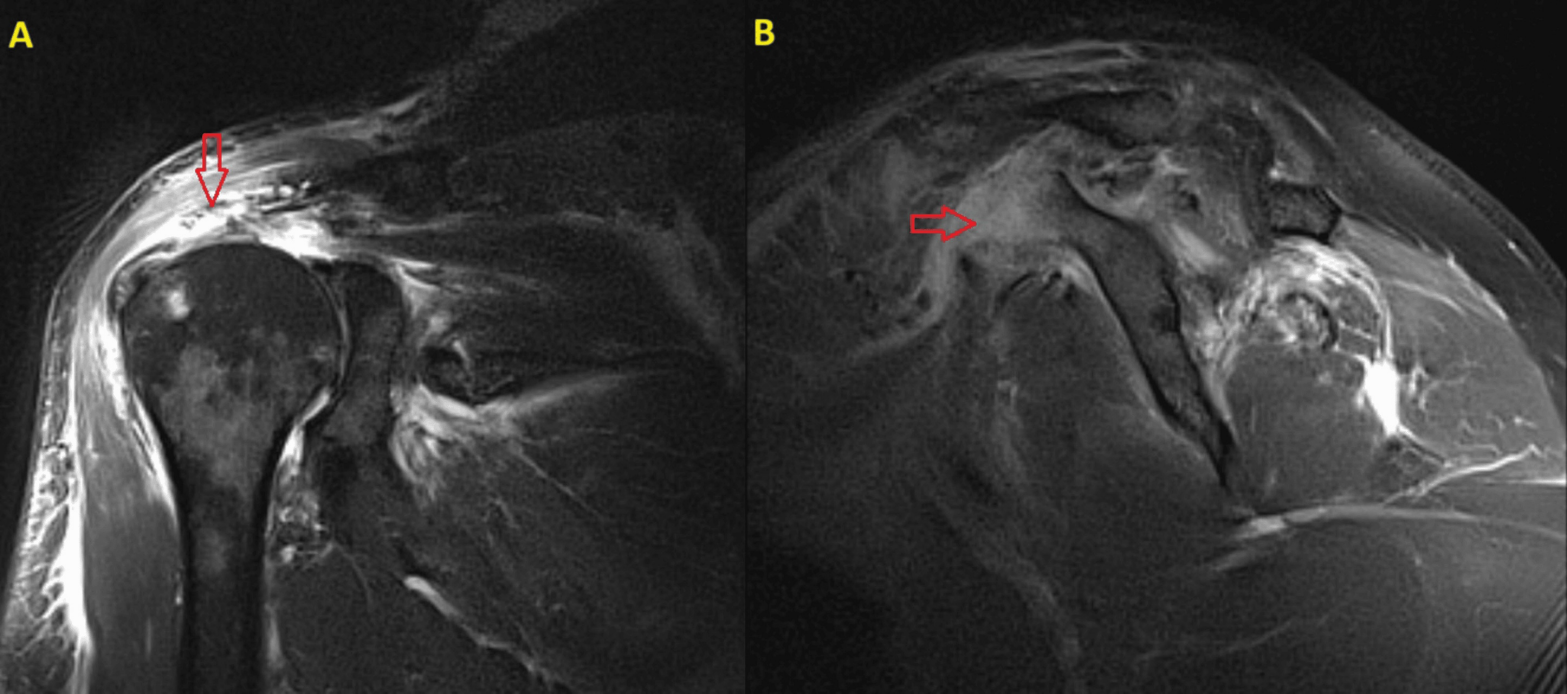
Preoperative MRI. (A) Coronal view: The red arrow indicates the rotator cuff tear. (B) Sagittal view: The red arrow demonstrates the coracoid process fracture.

A comprehensive physical examination revealed an inability to elevate the right upper extremity, suggesting possible shoulder damage. No neurovascular deficits were identified. Moreover, no other clinically evident injury was observed. Suspicion of a coracoid process fracture was initially raised on X-rays, but the diagnosis was confirmed with CT scan (Figures 1, 2). To evaluate for potential associated soft tissue injuries, MRI was performed the following day, confirming the presence of a massive rotator cuff tear (Figure 3).

In the operating theatre, the patient was placed in the beach chair position under general anesthesia. ORIF of the coracoid process was performed first, using a deltopectoral approach for direct access to the coracoid process. The fracture fragments were reduced and stabilized using bone reduction forceps, followed by internal fixation with a 44 mm (length), 4.5 mm (diameter) cortical lag screw and a washer to ensure optimal stability.

Subsequently, a second mini-open lateral approach was employed for rotator cuff repair. Intraoperatively, a full-thickness tear of the supraspinatus tendon was confirmed, with complete absence of the tendon at the footprint. Due to the irreparable nature of the supraspinatus, attention was turned to the infraspinatus tendon, which was found to be torn and retracted, but with sufficient tissue quality to allow mobilization and reattachment. Thus, the infraspinatus was carefully mobilized and reattached after burring the articular cartilage at the site where the tendon could reach, in approximately 40 degrees of shoulder abduction using two 5.5 mm titanium suture anchors. Despite this, a portion of the humeral head at the cuff interval remained uncovered by rotator cuff tissue. The subscapularis and biceps tendons were found to be intact, with no signs of rupture or degeneration.

**Figure 4:**
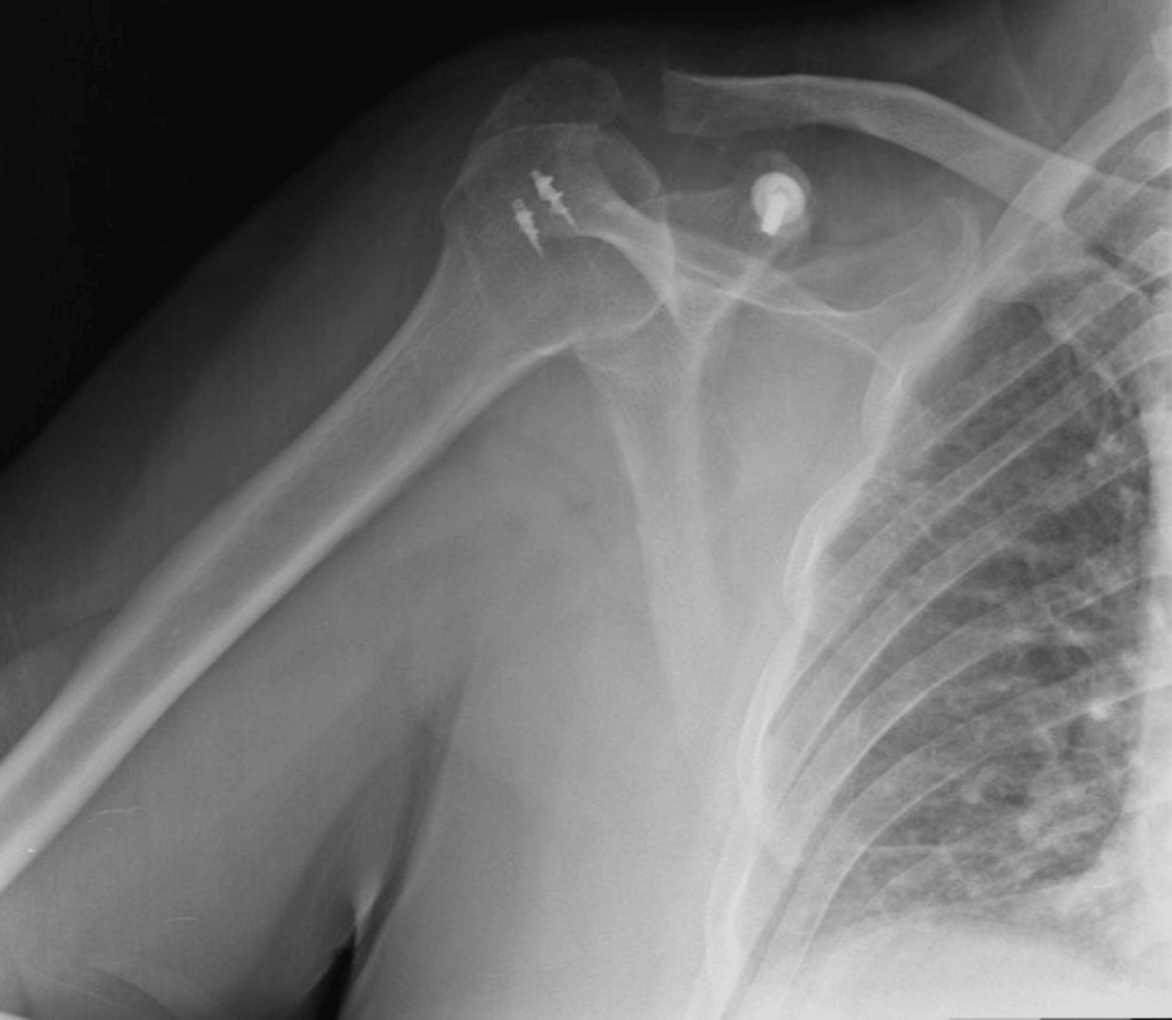
Postoperative one-month X-ray of the right shoulder joint displaying coracoid process fixation with a single screw and rotator cuff repair with two suture anchors.

Postoperatively (post-op), the patient was immobilized using a shoulder abduction pillow for six weeks. Passive range of motion, limited to the level of the brace, was initiated within the first 10 days after the repair. Afterwards, the patient followed a structured rehabilitation protocol, with active-assisted motion gradually introduced around six weeks post-op. Monthly clinical and radiological follow-up was conducted (Figure 4).

**Figure 5:**
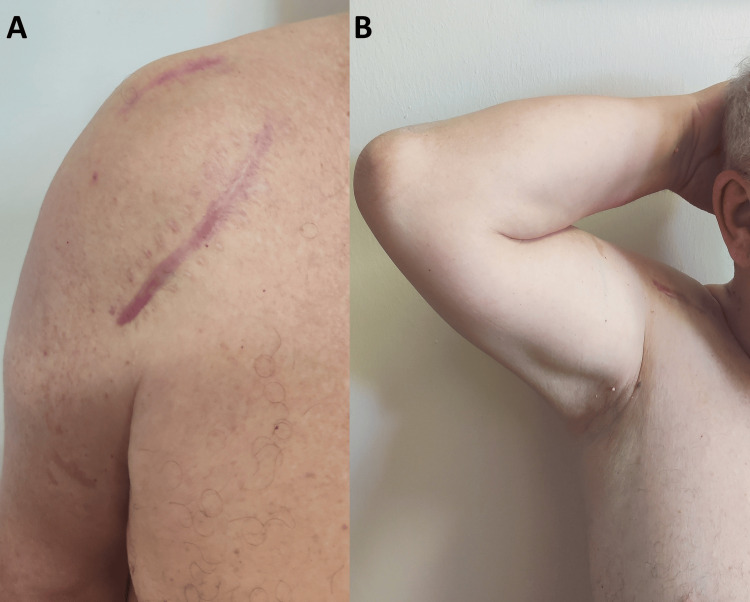
Postoperative clinical photographs at six months. (A) Anterior view of the right shoulder depicting the two well-healed surgical scars. (B) Anterior view of the right shoulder with the arm in abduction and external rotation, showing recovered range of motion.

**Figure 6:**
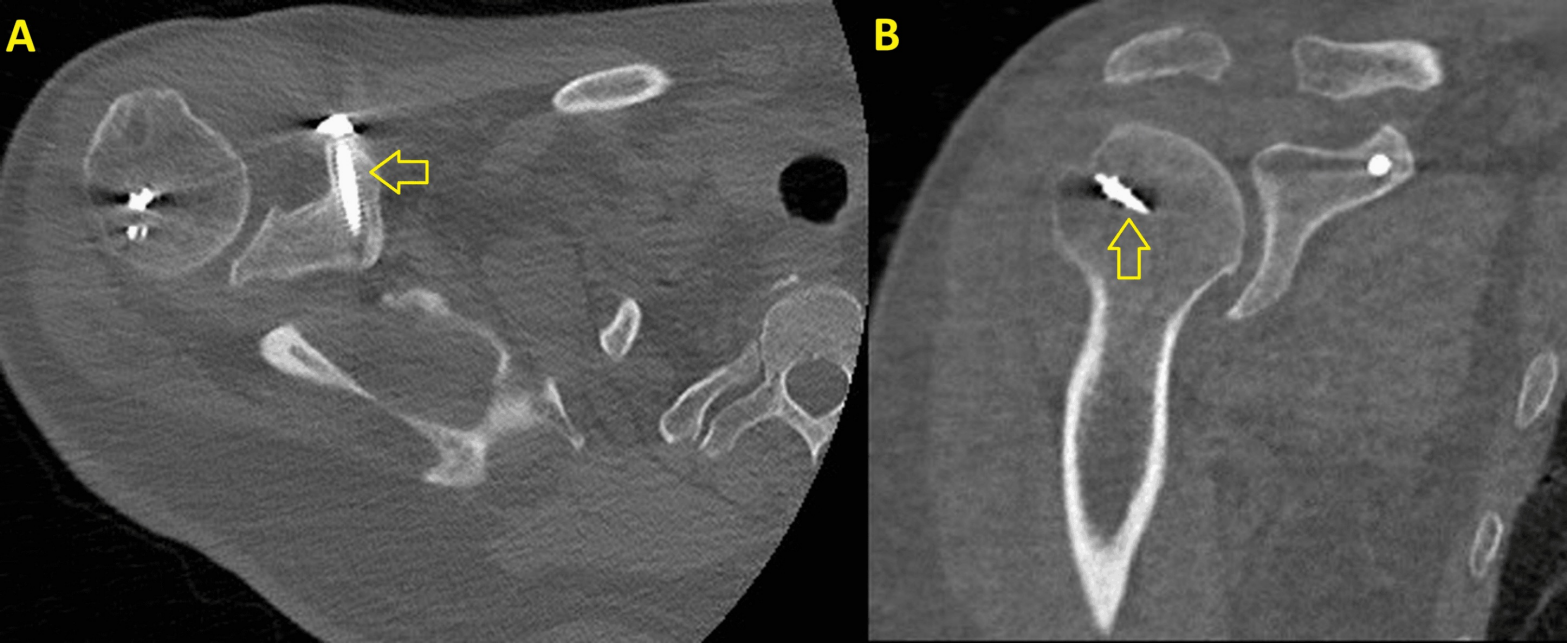
CT at six months postoperatively. (A) Transverse view: The yellow arrow points to cortical lag screw in the coracoid process. (B) Coronal view: The yellow arrow indicates one of the anchors used for rotator cuff repair.

Strengthening exercises were deferred until 12-16 weeks to ensure adequate tendon healing. At the four-month follow-up, the patient demonstrated gradual improvement in shoulder mobility with no signs of re-tear or postoperative complications. By six months, the patient had regained full range of motion in the right shoulder without pain and returned to his daily routine (Figure 5). A CT scan performed at that time revealed adequate ossification of the fracture site (Figure 6).

## Discussion

The present study highlights a rare and diagnostically challenging case involving a concomitant displaced coracoid process fracture and a massive rotator cuff tear, an unusual combination scarcely reported in the literature. Surgical management included open reduction and ORIF of the coracoid process using a single cortical screw via a deltopectoral approach. The rotator cuff tear was addressed through a mini-open lateral approach and repaired using a targeted partial reconstruction strategy.

To our knowledge, only Arun has reported a similar case involving a concomitant rotator cuff tear and coracoid process fracture in a 55-year-old man following a traffic accident [8]. However, that patient also sustained a shoulder dislocation with an anteroinferior labral tear, and a Hill-Sachs lesion. After diagnostic arthroscopic evaluation, the author employed a similar approach, using a deltopectoral incision for coracoid process fixation and a lateral approach for rotator cuff repair. The labral tear was not repaired, as the shoulder joint was found to be stable following the rotator cuff reconstruction.

Coracoid fractures typically result from high-energy mechanisms, such as direct trauma to the humeral head or avulsion forces from the muscles attached to the coracoid process, including the short head of the biceps brachii, coracobrachialis, and pectoralis minor [4,6]. These injuries are often underdiagnosed due to their deep anatomical location and nonspecific clinical presentation [3-5,9]. However, recent literature suggests an increasing incidence, underscoring the need for heightened clinical vigilance when evaluating shoulder trauma [6,9].

The mechanism of injury in our case, a direct anterior impact following a fall from height, suggests high-energy trauma consistent with the typical pattern seen in coracoid process fractures [2,4]. The patient's inability to elevate the arm, along with anterior shoulder pain, raised suspicion for both osseous and soft tissue injury. While initial radiographs were suggestive, they were inconclusive, necessitating further imaging for definitive diagnosis. As supported by prior literature, CT scan was essential for confirming the coracoid fracture and defining its morphology, while MRI was indispensable in identifying the full-thickness rotator cuff tear [2,4,5]. This highlights the importance of a multimodal imaging approach when clinical presentation is ambiguous or suggests multiple pathologies.

An important consideration in the management of coracoid fractures is the potential for associated neurological involvement. Coracoid process fractures have been reported to occasionally cause neurological complications, including brachial plexus compression and suprascapular nerve palsy [5,10]. In suspected cases, preoperative electromyography (EMG) can be helpful in assessing the extent of nerve involvement prior to surgical intervention [3,5,10]. However, no neurological deficits were observed in our case.

Regarding the management of coracoid process fractures, there are currently no universally accepted treatment guidelines [1]. Non-displaced fractures are generally treated conservatively [5,8,10]. Internal screw fixation is typically reserved for cases with displacement greater than 1 cm, symptomatic nonunion, associated ipsilateral scapular fractures, or injuries involving the superior shoulder suspensory complex [1,3,5,10]. The location of the fracture is also a key factor in treatment planning [3-5,8,10]. Fractures at the base of the coracoid, which may disrupt the coracoclavicular ligaments, often require operative intervention to prevent instability, whereas distal fractures can usually be managed nonoperatively [1,4,10].

While traumatic rotator cuff tears have been increasingly recognized in the context of high-energy trauma, especially in older patients, truly massive tears remain uncommon and are often underreported in the acute setting [4,7,11-14]. Milgrom et al. and Bassett and Cofield described traumatic rotator cuff tears involving full-thickness tendon avulsions with significant retraction as part of the "massive" tear spectrum, particularly when involving two or more tendons or >5 cm in size [11,12]. Early surgical intervention, either through open or arthroscopic techniques, is generally recommended within three weeks of injury for acute traumatic rotator cuff tears, as this timing has been associated with improved tendon healing and better functional outcomes [4,7].

In our patient, surgical management was undertaken not only for the displaced coracoid fracture but also for the massive rotator cuff tear that required repair. The deltopectoral approach for fixation of the coracoid process, combined with the mini-open lateral approach for rotator cuff repair, provided adequate surgical exposure that would have been difficult to achieve with a single approach, resulting in successful postoperative outcomes.

Interestingly, intraoperative findings revealed complete absence of the supraspinatus tendon. This, combined with involvement of the infraspinatus, fulfilled the criteria for a massive and partially irreparable tear. Given the non-viability of the supraspinatus tendon, our approach focused on mobilizing the intact infraspinatus. This tendon was reapproximated to a prepared humeral head footprint using two suture anchors, following burring of the articular cartilage. Despite this coverage, a segment of the humeral head at the cuff interval remained exposed post-repair. No supraspinatus release or tendon transfer was performed due to the patient’s tissue quality and intraoperative considerations. Alternative techniques such as superior capsular reconstruction were considered, but deemed unnecessary at this stage, given the integrity of the subscapularis, functional deltoid, and satisfactory tension-free repair of the infraspinatus.

Postoperative management in such complex injuries plays a pivotal role in achieving a favorable functional outcome. The structured rehabilitation timeline, which began with passive range of motion followed by gradual mobilization and delayed strengthening, was instrumental in promoting tendon healing while preventing stiffness. By six months, the patient had regained full, pain-free range of motion with radiographic evidence of fracture healing, underscoring the effectiveness of the surgical and rehabilitative strategy employed.

## Conclusions

This case emphasizes the importance of considering coracoid process fractures in the differential diagnosis of shoulder trauma, especially when accompanied by soft tissue injuries. A combined surgical approach addressing both bony and rotator cuff pathology can lead to favorable functional outcomes when followed by structured rehabilitation. Further reporting of similar rare cases will help refine diagnostic and treatment strategies for these complex injuries.
